# The complete mitochondrial genome of the Reef Manta Ray, *Mobula alfredi*, from Hawaii

**DOI:** 10.1080/23802359.2023.2167475

**Published:** 2023-02-02

**Authors:** Jonathan L. Whitney, Richard R. Coleman, Mark H. Deakos

**Affiliations:** a Pacific Islands Fisheries Science Center, National Oceanic and Atmospheric Administration, Honolulu, HI, USA; b Department of Marine Biology and Ecology, Rosenstiel School of Marine and Atmospheric Science, University of Miami, Miami, FL, USA; c Department of Integrative Biology, University of Texas, Austin, TX, USA; d Hawaiʻi Association for Marine Education and Research, Lahaina, HI, USA

**Keywords:** Reef Manta Ray, *Mobula alfredi*, Mobulidae, devil rays, mitogenome

## Abstract

We provide the complete mitochondrial genome of the reef manta ray, *Mobula alfredi*, using an ezRAD approach. The total length of the mitogenome was 18,166 bp and contained 13 protein-coding genes, 22 transfer RNAs genes, two ribosomal RNA genes, and one non-coding control region. The gene organization and length are similar to other *Mobula* species. This reference mitogenome that includes the control region is expected to be a valuable resource for molecular-based species identification, population genomics, and phylogeography.

## Introduction

Reef manta rays (*Mobula alfredi*, Krefft 1868) are emblematic inhabitants of coral reefs in tropical and subtropical oceans around the world (Last and Stevens 2009; Marshall et al. 2009). Many populations are in decline or may be threatened due to anthropogenic effects (Deakos et al. 2011; Croll et al. 2016; O’Malley et al. 2017; Stewart et al. 2018; Pate and Marshall 2020), which have contributed to their Vulnerable to Extinction status on the IUCN Red List of Threatened Species (Marshall et al. 2019). Despite their popularity and threatened status, our understanding of population structure remains limited. The whole mitogenome could be a powerful tool for population genomics of *M. alfredi*, as it offers high resolution assessment of gene flow and potential for exploring sex-based differences in behavior. However, no complete mitogenome reference is yet available for the species.

White et al. (2017) reclassified the family Mobulidae using mitogenomes, exons and morphology into a monophyletic genus, *Mobula*, with 8 accepted species. This reclassification included the placement of the genus *Manta* in synonymy with the genus *Mobula*, which was supported by prior phylogenetic analysis (Poortvliet et al. 2015). White et al. (2017) provided partial mitogenomes for most Mobulids, including *M. alfredi* (KX151653.1), however they were limited to the protein-coding genes and thus truncated before the control region. The control region (D-loop, displacement-loop) is non-coding and hypervariable and thus has value as an informative marker for evaluating gene flow across and population structure at fine spatial scales. Therefore, we sequenced the whole mitogenome of *M. alfredi*, including the control region, to provide a complete mitogenome reference valuable for application to species identification, population genomics and phylogeography of the reef manta ray.

## Materials and methods

**Figure 1.**
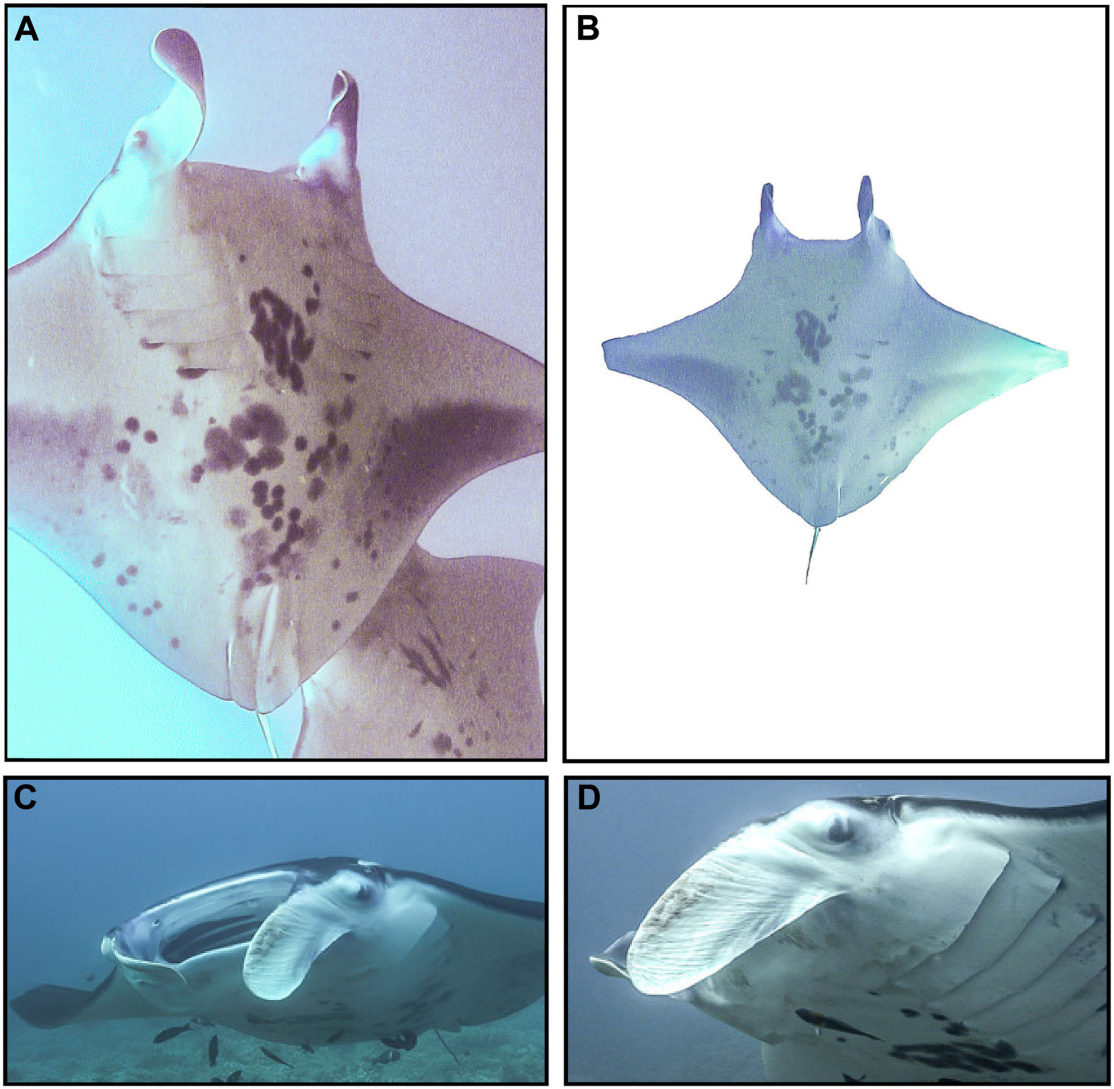
Voucher photographs (A–D) of wild adult female *Mobula alfredi* (HAMER CatalogID#176 ‘Bullseye’) sampled from Maui Island, Hawai’i, USA in November 2010.

Tissue samples of several *M. alfredi* individuals were obtained on 25 November 2010 off Olowalu Reef, Maui, Hawai‘i, USA (20.7913°N, 156.5880°W), including from the individual pertinent to this study, an adult female reef manta ray known as ‘Bullseye’ (Catalog #176) in the Hawaiʻi Association for Marine Education and Research photo-identification catalog (www.hamerinhawaii.org). Voucher photographs are provided in Figure 1. Biopsies (skin and muscle) were taken from the caudal end of the manta ray’s disk while on SCUBA using a modified Hawaiian sling containing a stainless-steel cylindrical biopsy tip (13 mm length, 5 mm diameter), preserved in 20% salt-saturated DMSO, and stored at −20 °C. Individuals are identified using unique ventral markings, gender and age-class are assigned based on clasper development in males (White et al. 2006; Marshall and Bennett 2010; Deakos et al. 2011) or mating scars and visible pregnancy in females (Marshall and Bennett 2010; Deakos 2012). Body size (disk width = 3.44 m) was measured using paired-laser photogrammetry as described in Deakos (2010). Tissues are deposited at the Pacific Islands Fisheries Science Center (PIFSC-MOALF-HAMR176-B19; contact Jonathan Whitney, Jonathan.Whitney@noaa.gov).

We extracted genomic DNA from tissue using an Omega E-Z 96 Tissue DNA Kit (Omega), following the manufacturer’s protocol. Due to the high prevalence of the GATC cut site in mitochondrial genomes as well as extensive random fragmentation in libraries, whole mitogenomes can be assembled from ezRAD libraries (Toonen et al. 2013; Tisthammer et al. 2016; Terraneo et al. 2018; Antaky et al. 2019), which simultaneously provide nuclear RAD loci for population genomics. We used the ezRAD approach (Toonen et al. 2013) to construct restriction-associated digest (RAD) reduced representation libraries with the enzyme DpnII (GATC cut site, New England Biolabs) following the ezRAD protocol (see Appendix 1 for detailed steps; Knapp et al. 2016). Genomic DNA was digested overnight with DpnII, end-repaired, 3′ ends adenylated, and ligated with Illumina TruSeq HT dual-indexed adapter sequences. DNA fragments from 300 to 425 bp (insert sizes 200–300 bp) were isolated using a PippenPrep (Sage Science). Adapter-ligated, size-selected fragments were amplified using PCR. Samples were cleaned using AMPureXP beads (Beckman-Coulter). DNA concentration was quantified using Accublue Quantitation (Biotium). A Bioanalyzer (Applied Biosystems) was used to check size-distribution of final amplified libraries. Libraries were normalized in equimolar concentrations (150 ng) and combined into pools with other libraries, bead cleaned and sequenced on one lane of an Illumina HiSeq 3000 (PE 150) at the UCLA Technology Center for Genomics and Bioinformatics. Raw reads were demultiplexed and only matched index pairs were retained.

**Figure 2.**
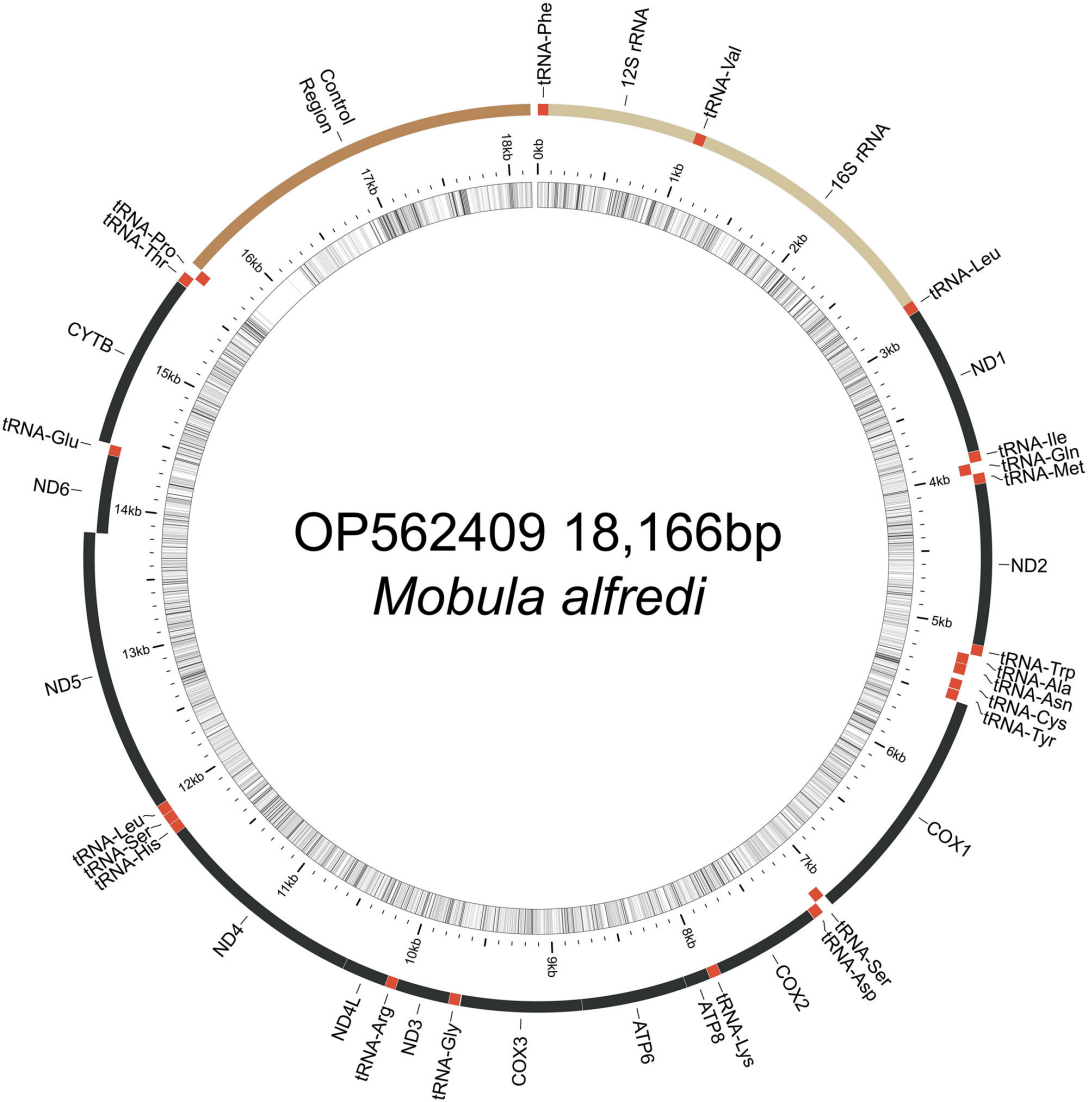
Map of the assembled *Mobula alfredi* mitochondrial genome (GenBank Accession: OP562409) consisting of 13 protein-coding genes (black), 22 transfer RNAs genes (red), two ribosomal RNA genes (light brown), and one non-coding control region (D-loop, dark brown). Genes encoded on the reverse strand and forward strand are illustrated inside the circle and outside the circle, respectively. The inner ring displays the GC content of the genome (every 5 bp), where the darker lines represent higher GC percent. This map was drawn using MitoAnnotator (Iwasaki et al. 2013).

We used the following workflow to assemble the *M. alfredi* mitogenome from ezRAD sequences. Raw reads were assessed using FastQC (Andrews 2010) and reads with adapters were filtered out using Cutadapt v1.11, (Martin 2011), and orphaned reads removed. We then used GetOrganelle v1.6.2d (Jin et al. 2020) to assemble mitogenomes *de novo* using the mitogenome of sister species *M. birostris* (KF413894 in Hinojosa-Alvarez et al. 2015) as the initial bait using all reads. The GetOrganelle assembly did not circularize but produced two long scaffolds totaling 18,097 bases. Scaffolds were aligned to 28 mitogenomes (4 complete, 24 partial) from 11 *Mobula* species available in GenBank (Table 1) using MUSCLE in Geneious Prime 2020 (www.geneious.com). This alignment revealed a 69-base gap in the 16S rRNA gene in our draft assembly, which we initially replaced with Ns to join scaffolds into a single 18,166 base assembly. To inform this gap, we extracted fresh gDNA from the same individual using a DNeasy Blood & Tissue Kit (Qiagen) and amplified a 587-bp fragment of the 16S rRNA gene using primers 16Sar-L:5′-CGCCTGTTTATCAAAAACAT-3′ and 16Sbr-H:5′-CCGGTCTGAACTCAGATCACGT-3′ (Palumbi et al. 1991). PCRs were performed in 20 μL reactions of 10 μL Immomix Red (Bioline), 0.5 μL BSA, 1.0 μL of each primer (1 μM), 5.5 μL water and 2 μL of gDNA with the following conditions: 95 °C for 5 min, followed by 35 cycles of 95 °C for 30 s, 50 °C for 30 s, 72 °C for 60 s, and a final extension of 72 °C for 10 min. Amplicons were purified with ExoSAP-IT (Thermo Fisher) and sequenced using an ABI 3730xl Sequencer (Applied Biosystems). The 16S sequences were aligned to the draft assembly using Geneious mapper with high sensitivity option. The 16S fragment was identical to overlapping sequence of the draft assembly and spanned the 69-base gap, generating a consensus sequence of the complete circular mitogenome (OP562409). The mitogenome was assembled from 27,434 paired Illumina reads and the 16S fragment, with mean coverage of 214× and high-quality base calls (% HQ) across 99.4% of the mitogenome. Gene annotation and validation of the circular mitogenome (Figure 2) was performed using MitoZ (Meng et al. 2019) and MitoAnnotator pipeline (Iwasaki et al. 2013). Pairwise sequence divergence was calculated between *M. alfredi* and sister species *M. birostris* (KF413894) using MEGA v.11 (Stecher et al. 2020, Tamura et al. 2021) on alignments of whole mitogenomes.

**Table 1.** Devil ray (Family Mobulidae) mitogenomes from Genbank.

Accepted species	Genbank accession	Complete/partial	Length	Genbank record description	Reference
*Mobula alfredi*	OP562409	Complete	18,166	Mobula alfredi mitochondrion, complete mitogenome	Current study
*Mobula alfredi*	KX151653	Partial	15,708	Manta alfredi isolate GN16688 mitochondrion, partial genome	White et al. 2017
*Mobula birostris*	KF413894	Complete	18,075	Manta birostris mitochondrion, partial genome	Hinojosa-Alvarez et al. 2015
*Mobula birostris*	KM364991	Partial	15,704	Manta birostris isolate MBI_MG03…complete sequence; mitochondrial	Poortvliet et al. 2015
*Mobula birostris*	KX151648	Partial	15,709	Manta birostris isolate GN6791 mitochondrion, partial genome	White et al. 2017
*Mobula hypostoma*	KX151646	Partial	15,719	Mobula hypostoma isolate GN5814 mitochondrion, partial genome	White et al. 2017
*Mobula hypostoma*	KM364992	Partial	15,662	Mobula rochebrunei isolate MRO_MG06… partial sequence; mitochondrial	Poortvliet et al. 2015
*Mobula kuhlii*	KM361353	Partial	15,716	Mobula eregoodootenkee mitochondrion, complete genome	Poortvliet et al. 2015
*Mobula kuhlii*	KM364987	Partial	15,715	Mobula kuhlii isolate MKU_MG10…complete sequence; mitochondrial	Poortvliet et al. 2015
*Mobula kuhlii*	KM364989	Partial	15,715	Mobula kuhlii isolate MKU_MG02 … complete sequence; mitochondrial	Poortvliet et al. 2015
*Mobula kuhlii*	KX151651	Partial	15,727	Mobula kuhlii isolate GN9426 mitochondrion, partial genome	White et al. 2017
*Mobula kuhlii*	KX151654	Partial	15,731	Mobula kuhlii isolate GN15461 mitochondrion, partial genome	White et al. 2017
*Mobula mobular*	JN184063	Partial	11,970	Mobula japanica mitochondrion, partial genome	Aschliman et al. 2012
*Mobula mobular*	JX392983	Complete	18,880	Mobula japanica mitochondrion, complete genome	Poortvliet and Hoarau 2013
*Mobula mobular*	KM364984	Partial	15,543	Mobula japanica isolate MJA_A03 … partial sequence; mitochondrial	Poortvliet et al. 2015
*Mobula mobular*	KM364988	Partial	15,544	Mobula japanica isolate MJA_L02 12S … partial sequence; mitochondrial	Poortvliet et al. 2015
*Mobula mobular*	KM435072	Partial	15,523	Mobula japanica mitochondrion, partial genome	Poortvliet et al. 2015
*Mobula mobular*	KM364983	Partial	15,711	Mobula mobular isolate MMO … complete sequence; mitochondrial	Poortvliet et al. 2015
*Mobula mobular*	KT203434	Complete	18,913	Mobula mobular mitochondrion, complete genome	Bustamante et al. 2016
*Mobula mobular*	KX151643	Partial	15,719	Mobula mobular isolate GN15654 mitochondrion, partial genome	White et al. 2017
*Mobula mobular*	KX151644	Partial	15,711	Mobula mobular isolate GN7058 mitochondrion, partial genome	White et al. 2017
*Mobula munkiana*	KM364990	Partial	15,717	Mobula munkiana isolate MMU_MG01…complete sequence; mitochondrial	Poortvliet et al. 2015
*Mobula munkiana*	KX151645	Partial	15,719	Mobula munkiana isolate GN5251 mitochondrion, partial genome	White et al. 2017
*Mobula tarapacana*	KM364986	Partial	15,713	Mobula tarapacana isolate MTA_MG05 … partial sequence; mitochondrial	Poortvliet et al. 2015
*Mobula tarapacana*	KX151647	Partial	15,730	Mobula tarapacana isolate GN10564 mitochondrion, partial genome	White et al. 2017
*Mobula tarapacana*	MH669414	Partial	15,686	Mobula tarapacana isolate CK02 mitochondrion, complete genome	Chandrasekaran et al. 2022
*Mobula thurstoni*	KM364993	Partial	15,719	Mobula thurstoni isolate MTH_MG08…complete sequence; mitochondrial	Poortvliet et al. 2015
*Mobula thurstoni*	KX151650	Partial	15,728	Mobula thurstoni isolate GN9728 mitochondrion, partial genome	White et al. 2017
*Mobula thurstoni*	MG206065	Complete	17,610	Mobula thurstoni mitochondrion, complete genome	Santillán-Lugo et al. 2017

## Results

The mitochondrial genome of *M. alfredi* (OP562409) is estimated at 18,166 bases in length including 13 protein-coding genes, 22 transfer RNAs genes, two ribosomal RNA genes, and one non-coding control region (Figure 2). Overall nucleotide composition was composed of 30.7% A, 29.8% T, 25.7% C, and 13.8% G. The gene organization and length are similar to other *Mobula* species, which range from 17,610 to 18,913 (Table 1). *Mobula alfredi* presents an AT-rich tandem repeat region in the control region, which varies in length and is found in other Mobulid rays (Hinojosa-Alvarez et al. 2015; Poortvliet et al. 2015; White et al. 2017). Pairwise sequence divergence between *M. alfredi* and sister species *M. birostris* was 0.009.

## Discussion and conclusions

**Figure 3.**
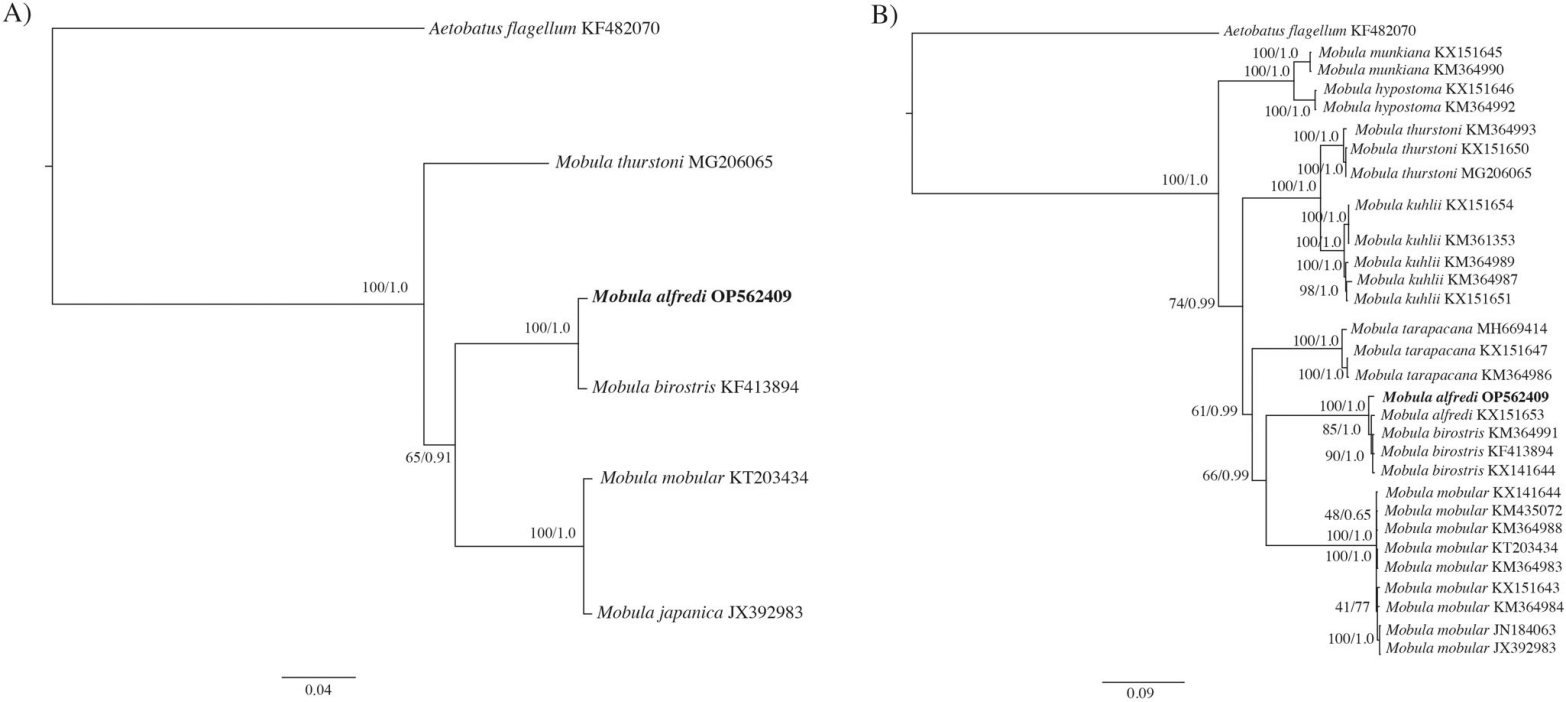
Molecular phylogenetic reconstruction of *Mobula*. Presented are rooted Bayesian trees based on (A) all available congeneric (*n* = 4) complete mitochondrial genomes (i.e. including the control region), and (B) all congeneric (*n* = 28) partial mitochondrial genomes (i.e. excluding the control region) (Table 1). Alignment and branch support analyses were performed in Geneious Prime v. 2022.0.1 The best fit sequence evolution model for both datasets, GTR + G (gamma = 0.2670, complete genome; gamma = 0.2650, partial genome), was identified by the Akaike Information Criterion using jModelTest v. 2.1 (Guindon and Gascuel 2003; Darriba et al. 2012). Phylogenetic reconstruction using a maximum likelihood (ML) analysis was created using PHYML v.3.0.1 (Guindon et al. 2010) as implemented in Geneious Prime with clade support assessed with 1000 non-parametric bootstrap replicates. Bayesian inference (BI) analysis was run using MrBayes v.2.2.4 (Huelsenbeck and Ronquist 2001; Ronquist and Huelsenbeck 2003) by running a pair of independent searches for 1 million generations, with trees saved every 1000 generations and the first 250 trees discarded as burn-in. Sequence divergence is represented on the scale bar. Branch support is presented as ML/BI. *Aetobatus flagellum* was selected as the outgroup. The resulting trees confirm the placement of OP562409 with *M. alfredi*, as sister to *M. birostris* and reinforces phylogenetic relationships established by previous studies (Poortvliet et al. 2015; White et al. 2017).

We present the first complete mitochondrial genome, including the control region, for *Mobula alfredi*. The complete genome phylogenetic tree (Figure 3(A)) confirms the placement of OP562409 as sister to *M. birostris* and within the genus *Mobula*. The partial genome tree (excluding the control region) confirms the placement of OP562409 within the *M. alfredi* species clade (Figure 3(B)). The divergence observed in the mitogenome between *M. alfredi* and *M. birostris* (*d* = 0.009) is similar to interspecific divergence rates that are suitable for species delineation among *Mobula* species (White et al. 2017). This complete circular reference mitogenome from the Hawaiian Islands includes the control region and is expected to be valuable for molecular-based species identification, population genomics, and phylogeography.

## Supplementary Material

Supplemental Material

## Data Availability

The genome sequence data that support the findings of this study are openly available in GenBank of NCBI at [https://www.ncbi.nlm.nih.gov] under the accession no. OP562409. The associated BioProject, SRA, and BioSample accession numbers are PRJNA899543, SRR22233901, and SAMN31097093 respectively.
