# Supplementary material for: The complete mitochondrial genome of the Reef Manta Ray, Mobula alfredi, from Hawaii
Source: Mitochondrial DNA B Resour. 2023 Feb 2;8(2):197–203. doi: 10.1080/23802359.2023.2167475 (PMC9901431; doi:10.1080/23802359.2023.2167475)
Supplement: Supplemental Material [file TMDN_A_2167475_SM0255.docx]

**Appendix**

for

**The complete mitochondrial genome of the Reef Manta Ray, *Mobula alfredi*, from Hawaii**

Jonathan L. Whitney^1*^, Richard R. Coleman^2,3^, and Mark H. Deakos^4^

^1^ National Oceanic and Atmospheric Administration, Pacific Islands Fisheries Science Center, Honolulu, Hawaiʻi

^2^ Department of Marine Biology and Ecology, Rosenstiel School of Marine and Atmospheric Science, University of Miami, Miami, Florida

^3^ Department of Integrative Biology, University of Texas, Austin, Texas

^4^ Hawaiʻi Association for Marine Education and Research, Lahaina, Maui

^*^Corresponding author. Email: Jonathan.Lyon.Whitney@gmail.com

With-Bead Library prep Protocol using Kapa HT TruSeq Kit (1/2 volumes)

(modified from Faircloth et al. 2014 – Illumina Prep v2.1 (BadDNA.org)

1. Concentrate genomic DNA (up to 3 ug) to 40 uL in centrivac (add H2O if <40 uL)

**Restriction Enzyme Digest**

1. Assemble RE digest master mix:

| 1X DpnII Buffer | 5 μL |
| --- | --- |
| DpnII (20 units) | 2 μL |
| Purified gDNA | 43 μL |
| **Total** | **50 μL** |

1. Add **7 uL digest master mix** to 43 uL gDNA and mix by pipetting (50 uL total volume).
2. Incubate in Thermocycler overnight (16hr) at 37 °C, then inactivate at 65 °C for 20 minutes, hold at 4 °C ∞
3. Run 1 uL of sample on gel (2%, 100 V for 55 min) to check digestion worked.

-------------------------------------------------SAFE STOPPING POINT-----------------------------------------------------------

**Post-digestion Cleanup**

1. Add **150 uL AmpureXP Beads** (3x Volume) to 50 uL digested DNA and mix by vortezing (200 uL total volume).
2. Conduct Ampure Cleaning Protocol.
3. Resuspend beads with **42.5 uL ddH2O** and **proceed immediately** to End Repair.

**End Repair**

1. Assemble the end-repair master mix:

| 10X End Repair Buffer | 5 μL |
| --- | --- |
| End Repair Enzyme Mix | 2.5 μL |
| **Total** | **7.5 μL** |

1. Add **7.5 uL** **End-repair master mix** to 42.5 uL bead/water/DNA solution and mix gently by pipetting (50 uL total volume).
2. Incubate in Thermocycler for 30 min @ 20 °C; hold at 10 °C or 4 °C for longer.

-------------------------------------------------SAFE STOPPING POINT-----------------------------------------------------------

**Post End Repair Cleanup**

1. Add **85 uL PEG (**1.7X) solution to 50 uL end-repair product and mix by pipetting or gently vortexing (135 uL total volume).

(Note: Bird uses 1.5X volume)

(PEG solution = 20% Polyethylene glycol (MW 8000), 2.5 M NaCl).

1. Conduct Ampure Cleaning Protocol and **proceed immediately** to A-tailing reaction.

**A-tailing Reaction**

1. Assemble the A-tailing reaction mix:

| 10X A-tailing Buffer | 2.5 μL |
| --- | --- |
| A-tailing Enzyme | 1.5 μL |
| ddH2O | 21 μL |
| **Total** | **25 μL** |

1. Add **25 uL A-tailing** **master mix** to dried beads and mix gently by pipetting.
2. Incubate in Thermocycler for 30 min @ 30 °C.
3. Add **45 uL PEG** solution (1.8X) to 25 uL reaction with beads and mix by pipetting or gently vortexing. (80 uL total volume).
4. Conduct Ampure Cleaning Protocol and **proceed immediately** to adapter ligation.

**Adapter-Ligation**

1. Assemble the adapter ligation master mix:

| 5X Ligation Buffer | 5 μL |
| --- | --- |
| DNA Ligase | 2.5 μL |
| ddH2O | 15 μL |
| **Total** | **22.5 μL** |

1. Add **22.5 μL ligation master mix** to dried beads and mix gently by pipetting.
2. Add **2.5 uL sample-specific Adapter* Mix** (30 uM) to the 22.5 uL bead/ligation mix solution and mix gently by pipetting.
3. Incubate for 15 min @ 20 °C, and proceed immediately to cleanup.

*Adapter Sequences

Illumina TruSeq HT dual-indexed adapters

GATCGGAAGAGCACACGTCTGAACTCCAGTCACNNNNNNNNATCTCGTATGCCGTCTTCTGCTTG

GATCGGAAGAGCGTCGTGTAGGGAAAGAGTGTNNNNNNNNGTGTAGATCTCGGTGGTCGCCGTATCATT

**Post-Ligation Cleanup**

1. Add **25 uL PEG** (1.0X) mixture to 25 uL ligation product and mix gently by pipetting or vortexing (50 μL total volume). We’re using less PEG solution at this step to help us remove very short fragments.
2. Conduct Ampure Cleaning Protocol.
3. Resuspend dried beads in **35 uL ddH20** and mix by pipetting. We will clean these again.
4. Add **35 uL PEG** solution (1.0X) mixture to reaction with beads. Mix by pipetting or gently vortexing. We’re using less PEG solution at this step to help us remove very short fragments.
5. Conduct Ampure Cleaning Protocol.
6. After drying, resuspend beads in **35 uL elution buffer (10 mM Tris-Acetate, pH 8)**, and incubate at room temp for 2-5 mins to release DNA from beads.
7. Capture beads by placing on magnet at room temp for 3-5 minutes or until solution is clear.
8. Recover the DNA in **35 μL** of supernatant and transfer to the tube/well and store at -20 °C.
9. Perform second elution on remaining beads in 35 uL elution buffer and store @ -20 **°**C for troubleshooting.

-------------------------------------------------SAFE STOPPING POINT-----------------------------------------------------------

**Size-Selection**

1. Use Pippen Prep to conduct size selection (30 μL input results in 40+ μL of size-selected library).

**Amplification**

1. Quantify 2 μL using Accublue (or QuBit)
2. Centrivac samples from 40 uL to 20 uL – and use half (10ul) for amplification.
3. Assemble the PCR master mix:

| 2X Kapa HiFi HotStart Master Mix | 12.5 μL |
| --- | --- |
| Primer Mix (2.5 uM each F + R primers) | 2.5 μL |
| **Total** | **15 μL** |

1. Add **15 uL PCR master mix** to **10 uL Adapter-ligated, size-selected DNA** (25 uL total volume)
2. Cycle using the following. You may need to adjust or optimize cycle number (6-8)

• 98 C for 45 seconds

• 6-12 cycles of: 98 C for 15 seconds; 60 C for 30 seconds; 72 C for 60 seconds

• 72 C for 5 minutes

**Final Cleaning of PCR Product**

1. Add **45 uL AMPure XP beads** (1.8X) to the 25 uL PCR product. Mix by pipetting or vortexing.
2. Conduct Ampure Cleaning Protocol.
3. Resuspend dried beads in **24 uL elution buffer (10 mM Tris-Acetate, pH 8)**, incubate at room temp for 2-5 mins to elute DNA.
4. Capture beads by placing on magnet at room temp for 3-5 minutes or until solution is clear.
5. Pull off **24 μL** of supernatant DNA and transfer to a new tube/well. This is the “pure gold” adapter-ligated, size-selected, amplified and cleaned DNA library to go to qPCR/sequencing.
6. Perform second elution on remaining beads in 30 uL elution buffer and store @ -20 **°**C for troubleshooting.

**Ampure Cleaning Protocol**

1. Incubate the mixture for 5 mins.
2. Move tubes to magnet & let sit for 3 mins (until solution is clear).
3. Aspirate liquid from tubes and discard (save supernatant for troubleshooting).
4. Add 200 uL 80% EtOH to each tube in the stand and incubate 30 seconds
5. Aspirate liquid from tubes and discard.
6. Add 200 uL 80% EtOH to each tube in the stand and incubate 30 seconds
7. Aspirate EtOH from tubes and discard.
8. Allow beads to dry for ~ 5 minutes or until there is no longer a smell of EtOH.
